# Multi-scale characterizations of colon polyps via computed tomographic colonography

**DOI:** 10.1186/s42492-019-0032-7

**Published:** 2019-12-27

**Authors:** Weiguo Cao, Marc J. Pomeroy, Yongfeng Gao, Matthew A. Barish, Almas F. Abbasi, Perry J. Pickhardt, Zhengrong Liang

**Affiliations:** 1 The Department of Radiology, Stony Brook University, Stony Brook, NY 11794 USA; 2 The Departments of Radiology and Biomedical Engineering, Stony Brook University, Stony Brook, NY 11794 USA; 3 The Department of Radiology, School of Medicine, University of Wisconsin, Madison, WI 53792 USA

**Keywords:** Colon cancer, Computed tomographic colonography, Polyp characterization, Texture feature

## Abstract

Texture features have played an essential role in the field of medical imaging for computer-aided diagnosis. The gray-level co-occurrence matrix (GLCM)-based texture descriptor has emerged to become one of the most successful feature sets for these applications. This study aims to increase the potential of these features by introducing multi-scale analysis into the construction of GLCM texture descriptor. In this study, we first introduce a new parameter - stride, to explore the definition of GLCM. Then we propose three multi-scaling GLCM models according to its three parameters, (1) learning model by multiple displacements, (2) learning model by multiple strides (LMS), and (3) learning model by multiple angles. These models increase the texture information by introducing more texture patterns and mitigate direction sparsity and dense sampling problems presented in the traditional Haralick model. To further analyze the three parameters, we test the three models by performing classification on a dataset of 63 large polyp masses obtained from computed tomography colonoscopy consisting of 32 adenocarcinomas and 31 benign adenomas. Finally, the proposed methods are compared to several typical GLCM-texture descriptors and one deep learning model. LMS obtains the highest performance and enhances the prediction power to 0.9450 with standard deviation 0.0285 by area under the curve of receiver operating characteristics score which is a significant improvement.

## Introduction

Colorectal carcinoma (CRC) is one of the top fatal diseases in the United States. American Cancer Society ranks CRC as the third most common cancer and the third leading cause of cancer-related deaths in both men and women [1]. There are two main categories of polyps, non-neoplastic and neoplastic. In general, the larger a polyp, the greater the risk of cancer is, especially with neoplastic polyps. Therefore, early polyp screening could effectively reduce the incidence of CRC [2, 3]. Computed tomographic colonography (CTC) is a minimally-invasive, cheap and safe screening method for polyps. However, subtle lesion diagnosis from these CTC images is still very challenging even for radiologists [4–6]. Nevertheless, computer-aided diagnosis (CADx) via tumor heterogeneity has shown great potential to handle this challenge [7–9].

**Fig. 1:**
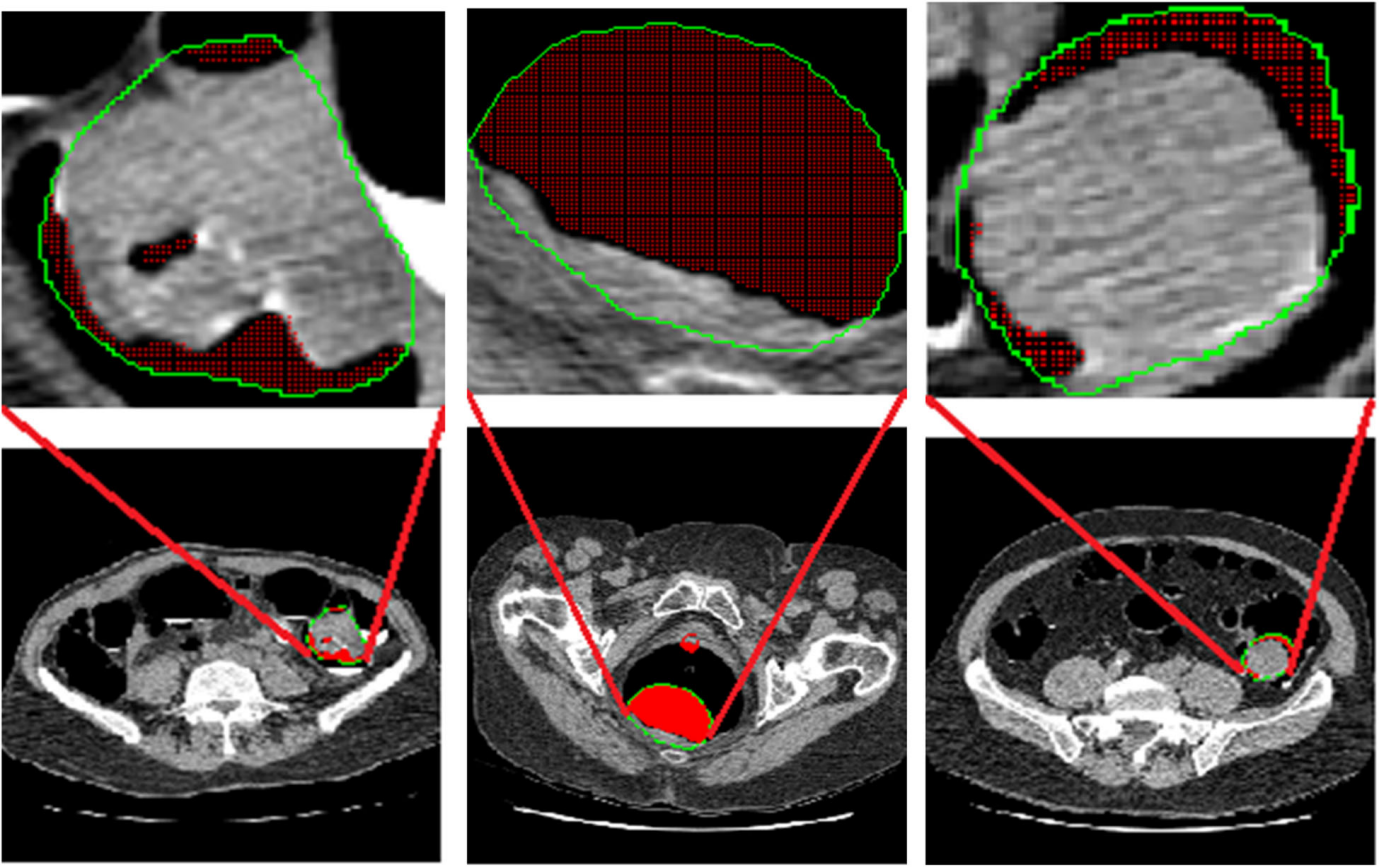
Polyp heterogeneity and texture in computed tomographic colonography. The green curves are their boundaries plotted by radiologists. The air in polyp is labeled by red color

Tumor heterogeneity describes the observation that different tumor cells can show distinct morphological and phenotypic profiles. It has become a critical measure in benign and malignant differentiability. The lesion’s heterogeneity is closely related to the lesion image textures (Fig. 1**)**. However, texture pattern extraction remains a great challenge [10–14]. The method proposed by Haralick et al. [15], the gray-level co-occurrence matrix (GLCM)-based texture descriptor, is identified as a promising solution for this problem. GLCM-based textures have been a forerunner in this field and adapted to multiple diseases such as polyps, breast cancer, lung nodules, gliomas, bladder cancer, and imaging modalities including CT, magnetic resonance imaging, positron emission computed tomography [16–19]. In the past, Lam [20] extended gray level co-occurrence matrix (CM) by gradient magnitude to extract image textures and Guo [21] explored CM by Gaussian curvatures to construct shape descriptors. In the recent years, Song et al. [22] introduced some high order metrics, such as gradient magnitude and curvature, to expand Haralick features (HFs) in volumetric data for polyp classification. To further improve the distinctions of the Haralick measures in different directions, Hu et al. [23] used the Karhunen-Loeve transform (KLT) to map the Haralick measures into an orthogonal eigenspace.

The Haralick model defines and extracts some important texture patterns from images. These patterns reveal image intensity correlation for pixel pairs on each two-dimensional (2D) image slice. Nevertheless, descriptors computed using the Haralick model in the 2D presentation have certain limitations. The model analyzes the nearest neighboring pixel in four different directions which is described in Section 2. The HFs are often extracted to construct rotational invariant descriptors which are formed by the means and ranges of Haralick measures along those four directions. However, the potential drawbacks of four-directional-averaging in a 2D digital image lack rotational robustness. On the other hand, the traditional Haralick model always counts all pixel pairs and calculates their distribution over all slices by full sampling which could result in redundant information and weaken the model’s performance. The third shortcoming of the Haralick model is the consideration of the nearest neighboring pixel to construct texture features: not considering other displacements may limit the potential to further extract textural patterns.

In this paper, we modify the definition of GLCM by adding a new variable - stride, and introducing multiple scaling analysis into the texture descriptor construction via GLCM. To address the weaknesses of the Haralick model, three schemes associated with each of the variables in GLCM, i.e., displacement, stride and angle, are devised to evaluate the CM-texture descriptors. Each scheme seeks to increase texture patterns through multiple scaling analysis while being mindful of texture information redundancy associated with the learning method. Furthermore, we intend to find out which variable would be more sensible in a multi-scale framework. Six classification schemes are designed for our investigation by random forest (RF).

The remainder of this paper is organized as follows: Section 2 describes and reviews the baseline Haralick model and proposes our new adaptive sampling model. Section 3 includes the analysis on the design and the results of our method. The last section includes some discussions and conclusions.

## Methods

This section begins with a review of the basic Haralick model in 2D and 3D space. Then, the proposed multiple scaling gray-level co-occurrence model (MSGLCM) is presented. The individual parameters from MSGLCM are each evaluated independently in three learning models, namely by multiple displacements, multiple strides and multiple angles.

### 2D/3D Haralick model

**Fig. 2:**
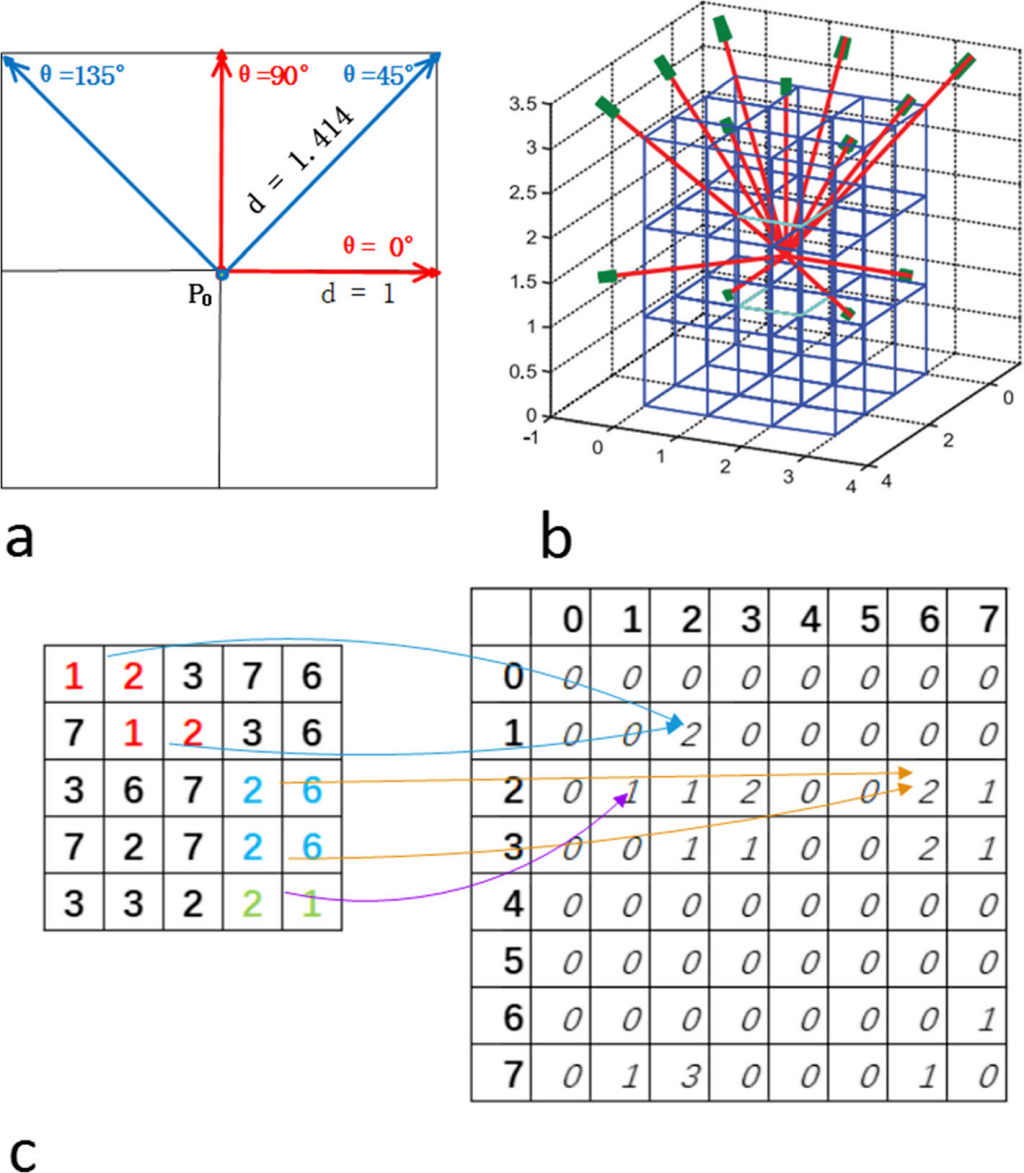
Illustration of co-occurrence matrix of two-dimensional images. **a**: Two-dimensional GLCM calculation; **b**: Three-dimensional GLCM calculation; **c**: A GLCM example when angle(θ) = 0°, displacement = 1. GLCM: Gray-level co-occurrence matrix

The Haralick model was proposed to extract polyp texture information from intensity images because of its strong ability to discriminate polyp pathologies [22, 23]. This model’s pipeline includes calculating image metrics such as its intensity, gradient and curvature, etc., image metric digitalization, GLCM computation, Haralick measure and feature definition, and image descriptor construction. The GLCM computation defines and extracts important texture patterns (distribution of pixel-pairs) from one image along different directions (Fig. 2).

The method provides 14 measures for every matrix computation. In a 2D gray image, four directions (0°, 45°, 90° and 135°) are analyzed (Fig. 2a). From each direction, one image would generate HFs consisting of 28 texture variables, i.e., 14 means and 14 ranges which would be used to construct the texture descriptor. In contrast, the number of directions in volumetric data is 13 (Fig. 2b). Hu et al. [23] expanded this model to generate 30 measures, referred to as the extended Haralick measures (eHM), to capture more texture information from volumetric data. Unlike the Haralick model, they employ all measures to form the texture descriptor instead of HFs.

### Proposed multi-scaling GLCM model

The proposed method utilizes three primary variables for the multi-scaling model, i.e., displacement scaling, stride scaling, and angle scaling. Using these values, the equation for the multi-scaling GLCM (MSGLCM) can be presented as below:

1 $$ {C}_{i,j}\left(d,\theta, s\right)={\sum}_{\begin{array}{c}m=1,\\ {}m\leftarrow m+s\end{array}}^M{\sum}_{\begin{array}{c}n=1\\ {}n\leftarrow n+s\end{array}}^N\left\{\begin{array}{cc}1& I\left(m,n\right)=i,I\left(\left(m,n\right)+\mathrm{d}\ast \uptheta \right)=j\\ {}0& \mathrm{otherwise}\end{array}\right. $$

**Fig. 3:**
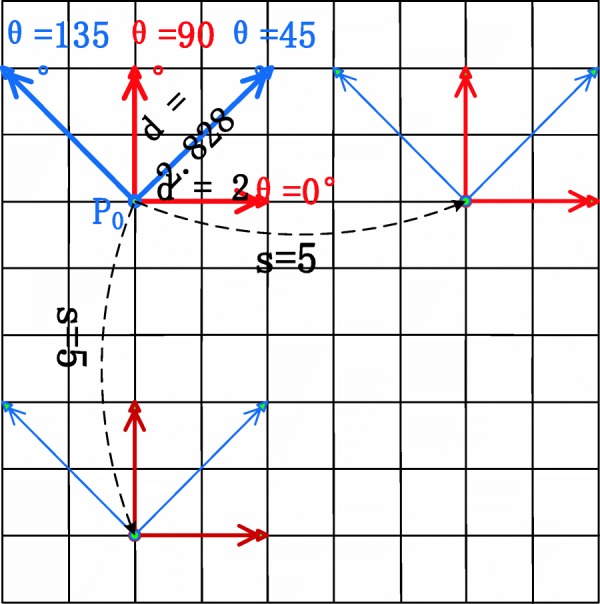
Calculation of multi-scale gray level co-occurrence matrix where *d* represents displacement, *θ* is the angle, *s* is stride (or scale), *p*_*0*_ is the concerned point

*I* represents the grayscale image, (*M*, *N*) is the image size, *i* and *j* are a pair of image pixel values, *d* is the displacement between two pixels along the angle *θ*, and *s* represents the stride. A pictorial illustration of Eq. (1) is shown in Fig. 3. When *s* = *d* = 1, the MSGLCM should be the traditional GLCM model. This model provides a new tool to capture more texture patterns at multiple scales. A typical example of MSGLCM calculation is shown by Fig. 3 where the stride is equal to 5. The MSGLCM model for 3D volumetric data is similar to the 2D model except that its coefficients are bidirectional.

According to MSGLCM definition, there are three important variables in the learning model, i.e., displacements, strides and angles. Each variable would be investigated individually and expanded to larger magnitudes to determine their individual behavior in the model. The following subsections present the methods where each of these three parameters are investigated for the contribution to the multi-scaling framework.

#### Learning model by multiple displacements

The traditional Haralick model has a sampling distance of 1. In medical images, there are more complex textures and using a displacement of 1 might limit the information used to define texture patterns. To evaluate the effect of displacement on the texture pattern, the other two coefficients, i.e., angle and stride, are fixed as follows:

2 $$ {C}_{i,j}^{MD}(d)={\sum}_{\begin{array}{c}m=1,\\ {}m\leftarrow m+1\end{array}}^M\ {\sum}_{\begin{array}{c}n=1,\\ {}n\leftarrow n+1\end{array}}^N\left\{\begin{array}{cc}1& I\left(m,n\right)=i\&I\left(\left(m,n\right)+d\ast {\theta}_0\right)=j\\ {}0& otherwise\end{array}\right. $$

where θ_0_ ∈ {(0, 0, 1), (0, 1, 0), (1, 0, 0), (0, 1, 1), (1, 0, 1), (1, 1, 0), (− 1, 1, 0), (0, 1, -1), (1, 0, -1), (1, 1, 1), (− 1, 1, 1), (1, 1, -1), (− 1, 1, -1)} as shown in Fig. 2b.

**Fig. 4:**
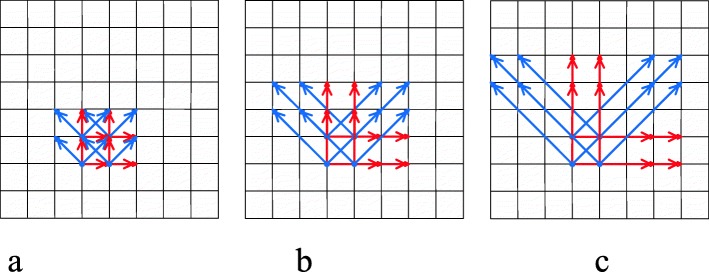
MSGLCM calculation by displacement samplings: (**a**) displacement = 1, (**b**) displacement = 2, (**c**) displacement = 3

In learning model by multiple displacements (LMD), we adopt an up-sampling method to get more texture patterns. Considering the small volumes of the polyps, large displacements are not ideal while calculating MSGLCM. Smaller displacements, i.e., 1, 2, 3, are used in this exploration study (Fig. 4).

The calculation produces three matrix sets for three displacements. Each matrix set contains 13 matrices associated with 13 digital angles [23]. This method generates more texture patterns and texture descriptors for polyp classification compared to the traditional Haralick model.

#### Learning model by multiple strides

With the increased information that can be extracted with the MSGLCM model compared to the traditional method, the stride can be used as a form of down sampling to control multiple scaling implements while calculating the CM. Suppose the current position is (x, y); the next position for the model would be (x + stride, y) in the row, or (x, y + stride) in the column. A similar technology can be found in deep learning [24, 25]. In this scheme, stride is the variable which is kept for evaluation while the displacement and angle are constants as described by the following equation.

3 $$ {C}_{i,j}^{MS}(s)={\sum}_{\begin{array}{c}m=1,\\ {}m\leftarrow m+s\end{array}}^M\ {\sum}_{\begin{array}{c}n=1\\ {}n\leftarrow n+s\end{array}}^N\left\{\begin{array}{cc}1& I\left(m,n\right)=i,I\left(\left(m,n\right)+{d}_0\ast {\theta}_0\right)=j\\ {}0& otherwise\end{array}\right. $$

where *d*_0_ is the fixed displacement and *θ*_0_ has 13 alternatives as shown in Formula (2).

**Fig. 5:**
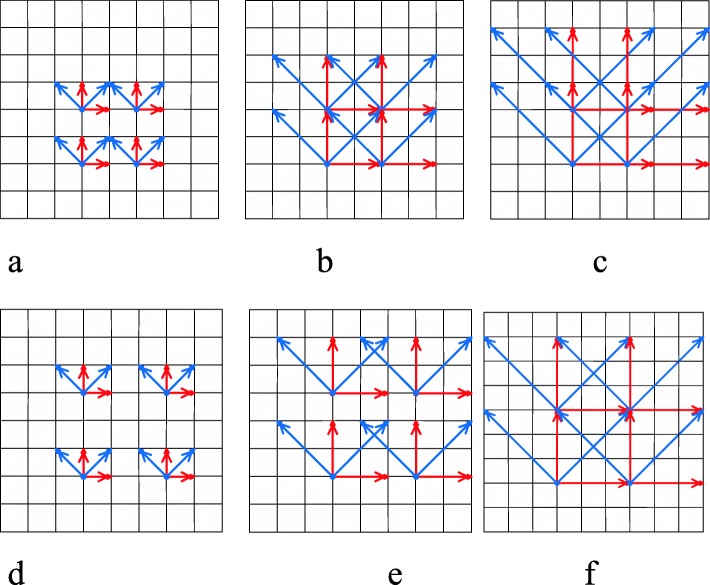
ASGLCM calculation on one slice of a volume, containing 13 directions in the 3D space: (**a**) stride = 2, displacement = 1, (**b**) stride = 2, displacement = 2, (**c**) stride = 2, displacement = 3, (**d**) stride = 3, displacement = 1, (**e**) stride = 3, displacement = 2, (**f**) stride = 3, displacement = 3

This method is similar to LMD with the addition of the stride analysis for the MSGLCM calculation. Unlike displacement, increasing the stride will lead to a down-sampling process. Likewise, smaller strides are considered more ideal for MSGLCM calculation since the sizes of the polyps are always small. The strides that are evaluated for this method will be limited by the size of the region of interest (ROI) volumes used. The base model for this design includes 13 directions and a displacement of 1, though it can be further expanded with LMD to increase the displacement and stride. Example cases of using a stride of 2 and 3 are illustrated in Fig. 5.

In image classification, the performance is significantly determined by some key features. The traditional full sampling will generate more redundancy while decreasing the ratio of key features, which will hurt the clustering performance. This method provides a solution via decreasing the sampling frequency over the image to lessen the number of non-critical features. Therefore, the down-sampling method intends to enhance the roles of key features in polyp classification to improve the clustering results.

#### Learning model by multiple angles

Angle sampling rate in a 3D image array can mitigate sparse directions in the model by including higher orders of neighbors in CM. The angles in digital images or volume data are discretized and as a result, increasing the digital angles requires more displacements in the digital domain. Similar to the previous designs, i.e., LMD and learning model by multiple strides (LMS), the displacements used to evaluate the new design are 1, 2 and 3 due to concern for polyp size (i.e., 3 mm and larger). The following equation describes the MSGLCM with variable angles and a fixed stride.

4 $$ {C}_{i,j}^{MA}\left(\theta \right)={\sum}_{\begin{array}{c}m=1,\\ {}m\leftarrow m+1\end{array}}^M\ {\sum}_{\begin{array}{c}n=1,\\ {}n\leftarrow n+1\end{array}}^N\left\{\begin{array}{cc}1& I\left(m,n\right)=i\&I\left(\left(m,n\right)+\theta \right)=j\\ {}0& otherwise\end{array}\right. $$

where θ is the digital angle represented by some 3D vectors similar to Formula (2).

**Fig. 6:**
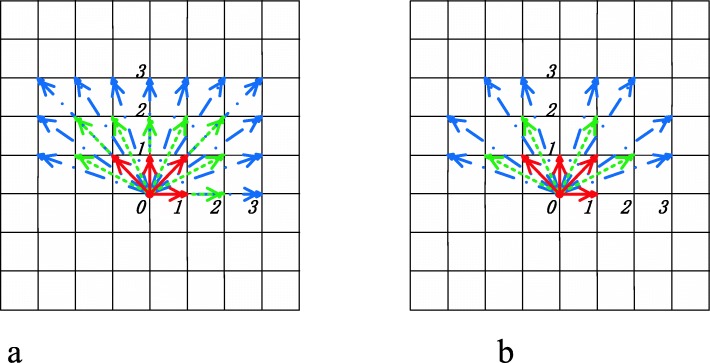
Two cases of multiple angle sampling: (**a**) multiple angle sampling with duplicates, (**b**) multiple angle sampling without duplicates

It is easy to see this is an up-sampling model similar to LMD. Furthermore, LMD is a subset of learning model by multiple angles (LMA). Each displacement could generate a set of angles (Fig. 6a). The angles of different displacements are listed in Table 1. To examine the behavior of multiple angles, the displacement and the stride will be set as 1 for the base model. Further observation will include increasing the displacement and increasing the stride. Note that some angles in digital images can be duplicated as we include more directions while increasing displacements (Fig. 6a). To investigate the impact of these repeated angles in polyp classification, they are removed in another scheme, as shown in Fig. 6b.

**Table 1:** Angle groups of two cases in Fig. 6 under three different displacements

Angles	Displacement	Displacement	Displacement
	≤1	≤2	≤3
With duplicates	13	62	171
Without duplicates	13	49	145

All the proposed models could be able to generate new texture information different from the traditional Haralick model via multi-scaling on displacements, strides and angles. However, with the increased pool of information, the texture patterns would bring not only more useful information but also some redundancies. This can potentially lead to overfitting problems in polyp categorization which could lower the clustering performance and consequentially hurt the classification. There are numerous debates on this topic which could be solved by appropriate feature selection methods [26–28].

### Polyp descriptors and classifier

#### Polyp descriptors

Polyp descriptors are numeric descriptions in the form of scalars, vectors, or matrices that describe a polyp extracted from a polyp image or volume. In this article, the eHM are utilized to construct the polyp descriptors [23]. For MSGCLM, eHM defines 30 measures that expands the 14 traditional Haralick measures with 16 new measures. However, the 21th measure which represents cluster average is always equal to 0, and the 25th and 30th measures are equivalent after formula simplification. Therefore, the descriptor will include 28 measures for one direction. For multiple angles, the vector will have N * 28 variables to represent a polyp where N is the angle number.

#### Classifier and feature selection

Classification is one of the most effective tools for identifying descriptors. Its major task is to identify general patterns belonging to one category. The simplest case is binary classification which creates a function *g* : *x* → {1, −1}, where *g* is a classifier [29].

RF classification is derived from a decision tree (DT) method [30]. Unlike DT, RF will apply many trees to train and test the samples, then a voting method is used to get the probability from these trees. Another distinction is the random sampling in the tree construction that includes randomly splitting features, combinations of features and choosing the threshold. For each process of RF, the descriptors of all polyps are divided into training groups and testing groups. Before classification, we first calculate the priority of each variable in the texture descriptor. GINI coefficient is introduced to be the priority measurement in our method. Then some variable sets are generated using the forward step feature selection method on the ranked variables [23, 31]. Thereafter, classifications are performed on each variable set under the parameter of 2000 trees and $\sqrt{N\ast 28}$ candidate variable number. We utilize the area under the curve of receiver operating characteristics (AUC) to be our evaluation measurement. The feature set with the highest AUC score would be taken to be the optimized texture descriptor.

### Some operations for volume of interests and digital angles

Before differentiating the types of polyps, each polyp’s position (x, y, z) in a volumetric data was labelled by radiologist experts. Next, a semiautomatic performance is adopted to crop the polyp patches on every image slice. For that purpose, the labeled polyps are outlined manually to generate ROIs on all slices according to the labelled location. The polyp locations are continuous: located on every slice and form a volume of interest (VOI). Due to the manual labelling, the resulting VOIs include additional information such as air. To separate the air from the polyp, an adaptive air-cleansing algorithm is employed to eliminate those voxels that contain predominately air [32].

The digital angles are defined in accordance to the grid structure of a digital image. As a result, the distance for each digital angle may not always be integers. To address this issue, vectors are used to provide information on angle and magnitude which correspond to the angle and displacement in the proposed models.

The traditional GLCM is calculated including the inverse angles to produce a symmetric matrix. The Haralick measures are symmetrically invariant; therefore, the matrix and its symmetric iteration can produce the same measures. To reduce redundant measures, the inverse directions are excluded from the digital angles. Only four angles are included in the 2D Haralick model corresponding to (1, 0), (1, 1), (0, 1), and (1, − 1). Similarly, the extended Haralick model in 3D would include more directions with the increase of the displacement.

## Results

### Polyp dataset

A private dataset containing 59 patients with a total number of 63 polyp masses is used for the experiments of this study. All the polyp masses are at least 30 mm in diameter. Each polyp was identified by radiologist experts on CTC and optical colonoscopy. All the patients were scheduled for surgical removal intervention after detection and confirmation. When the polyp masses were removed, all pathology reports were obtained to verify whether each of the polyp masses was indeed a cancerous (adenocarcinoma) or benign (adenomatous) polyp. The breakdown of the dataset can be seen in Table 2. To benefit surgical intervention, it is important to know the malignant risk of each polyp mass. Given the pathology reports, these polyp CTC scans provide an excellent database to develop machine learning strategies to predict adenocarcinoma for more aggressive removals. In addition to direct clinical impact, this database also provides good opportunities to evaluate different machine learning strategies regarding to pathological ground truth. This study is an example of evaluating methodology development for polyp classification using the pathologically approved database.

**Table 2:** Polyp masses dataset used for experiments

Category	Pathology	Count	Male: Female	Average size (mm)
Benign (0)	Serrated adenoma	3	2:01	34.3
	Tubular adenoma	2	2:00	35
	Tubulovillous adenoma	21	11:10	37.6
	Villous adenoma	5	4:01	55
Malignant (1)	Adenocarcinoma	32	12:20	43.9

Classification needs two sub-datasets, the training dataset and the testing dataset. From the polyp mass database, we randomly selected 15 samples from the benign polyps and 16 from the malignant polyps for training. The remaining polyps are used for testing. Thus 31 polyps are used for training and 32 for testing (Table 3). Repeating the random sampling method, we generated 100 unique iterations for training and testing groups.

**Table 3:** The training samples and testing samples for polyp classification

Dataset	Total	Category	Number
Training	31	Benign	15
		Malignant	16
Testing	32	Benign	16
		Malignant	16

### Experimental outcomes

According to the three multi-scale models, six testing schemes are designed. We test the three models separately. Then three hybrid experiments are designed and implemented.

#### Results of LMD, LMS and LMA

To calculate LMD, the displacements vary between {1, 2, 3} while its stride remains constant at 1 and with 13 angles. As we test LMS, its strides vary in {1, 2, 3, 4, 5, 6, 7, 8, 9}. Its displacement remains 1 and 13 angles are involved in the calculation. For LMA, both its displacement and stride stay 1 while the total angles are set as {13, 62, 171}. After classification, their AUC scores are listed in Table 4. Their results tell us that the stride is more effective to improve the descriptor distinction than the other two parameters of displacement and angle since its AUC score is improved by about 6%. Compared with eHM (baseline), the LMD and LMA are almost even and do not bring much gain for polyp classification when we change the displacement and the angle numbers independently.

**Table 4:**
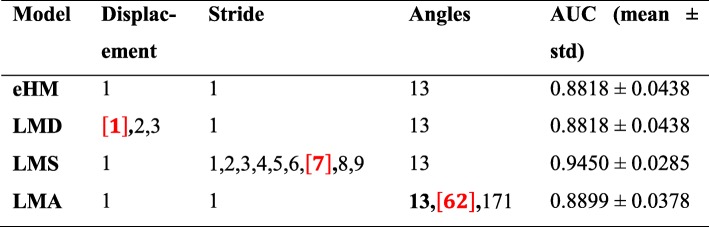
The OCR for LMD, LMS and LMA

The red number in square brackets represents the parameter for the OCR. *AUC* Area under the curve of receiver operating characteristic curve; *OCR* Optimized classification results, *eHM*: Extended Haralick measure, *LMD* Learning model by multiple displacements, *LMS* Learning model by multiple strides, *LMA* Learning model by multiple angles

#### Hybrid results of LMD + LMS

In this experiment, LMD and LMS are combined to extract some new texture patterns. There are 3 different displacements and 9 strides involved in texture descriptor construction. Hence, 27 kinds of polyp descriptors are generated. The number of angles is kept constant with 13 directions. After training and testing via RF, their AUC scores are calculated and illustrated in Table 5. It retells us that the stride is more sensitive than the displacement. With the stride increasing, we see that the classification performance generally increases in a staggering fashion. However, the trend of AUC scores on each row are gradually declining while the displacement is growing which means LMD introduces more redundant information for polyp classification.

**Table 5:** AUC scores of three CM sets with nine different strides for LMD + LMS where D represents displacement

	D = 1	D = 2	D = 3
S = 1	0.8818 ± 0.0438	0.8725 ± 0.0461	0.8706 ± 0.0489
S = 2	0.8621 ± 0.0487	0.8486 ± 0.0458	0.8751 ± 0.0477
S = 3	0.9043 ± 0.0382	0.8765 ± 0.0407	0.8786 ± 0.0463
S = 4	0.8919 ± 0.0382	0.8834 ± 0.0368	0.8907 ± 0.0417
S = 5	0.9159 ± 0.0408	0.8971 ± 0.0443	09073 ± 0.0446
S = 6	0.9057 ± 0.0394	0.8988 ± 0.0400	09042 ± 0.0447
S = 7	0.9450 ± 0.0285	0.9303 ± 0.0393	0.9223 ± 0.0375
S = 8	0.8887 ± 0.0366	0.8924 ± 0.0436	0.8838 ± 0.0397
S = 9	08825 ± 0.0452	0.9073 ± 0.0421	0.8714 ± 0.0402
Average	0.8975 ± 0.0.399	0.8896 ± 0.0421	0.8893 ± 0.0435

*AUC* Area under the curve of receiver operating characteristic curve, *CM* Co-occurrence matrix, *LMS* Learning model by multiple strides, *LMD* Learning model by multiple displacements

**Fig. 7:**
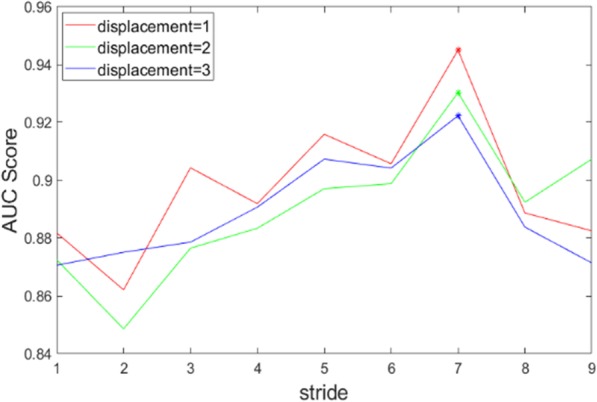
AUC score trends with the stride increasing for LMD + LMS. “*” indicates the best result position of each curve. AUC: Area under the curve of receiver operating characteristic curve; LMS: Learning model by multiple strides; LMD: Learning model by multiple displacements

From these results, there is an increasing trend with odd strides as the stride approaches 7. Similar outcomes are reached with the increasing displacements. These outcomes demonstrate that the LMA model with a larger stride could produce more critical features and get much better performance for polyp classification. Figure 7 is plotted to illustrate the AUC score changes according to stride and displacement.

#### Hybrid results of LMS + LMA

In this experimental scheme, we try to combine LMS and LMA to investigate the second hybrid model with three parameters. The angle sampling method in Fig. 6 shows that more digital angles need more displacements which determines the digital angle number under full sampling. That means angle group and displacement exists in a one-to-one relationship under a full sampling scheme in digital images. Therefore, this type of hybrid model contains two parameters, i.e., angle number and strides. Moreover, the previous schemes indicate that the displacement does not obtain any benefit, while the stride produced significant impact on the AUC score. Therefore, the following scheme keeps 3 displacements while the stride varies from one to nine. The results of the scheme with duplicate angles are described in Table 6 and the scheme without duplicate angles is described in Table 7.

**Table 6:** AUC scores of LMA with duplicates angles over 100 training and testing groups

	Angles = 13	Angles = 62	Angles = 171
S = 1	0.8818 ± 0.0438	0.8899 ± 0.0378	0.8887 ± 0.0377
S = 2	0.8621 ± 0.0487	0.8527 ± 0.0463	0.8561 ± 0.0512
S = 3	0.9043 ± 0.0382	0.9053 ± 0.0387	0.9055 ± 0.0374
S = 4	0.8919 ± 0.0382	0.9075 ± 0.0369	0.8993 ± 0.0354
S = 5	0.9159 ± 0.0408	0.9211 ± 0.0385	0.9246 ± 0.0347
S = 6	0.9057 ± 0.0394	0.90764 ± 0.0364	0.9093 ± 0.0392
S = 7	0.9450 ± 0.0285	0.9457 ± 0.0293	0.9378 ± 0.0304
S = 8	0.8887 ± 0.0366	0.9004 ± 0.0373	0.8959 ± 0.0371
S = 9	08825 ± 0.0452	0.9194 ± 0.0391	0.9187 ± 0.0435
Average	0.8975 ± 0.0399	0.9055 ± 0.0378	0.9039 ± 0.0385

*AUC* Area under the curve of receiver operating characteristic curve, *LMA* Learning model by multiple angles

**Table 7:** AUC scores of LMA without duplicate angles over 100 training and testing groups

	Angles = 13	Angles = 49	Angles = 145
S = 1	0.8818 ± 0.0438	0.8833 ± 0.0387	0.8926 ± 0.0383
S = 2	0.8621 ± 0.0487	0.8524 ± 0.0512	0.8515 ± 0.0499
S = 3	0.9043 ± 0.0382	0.9054 ± 0.0388	0.9062 ± 0.0387
S = 4	0.8919 ± 0.0382	0.9084 ± 0.0376	0.8918 ± 0.0385
S = 5	0.9159 ± 0.0408	0.9176 ± 0.0337	0.9263 ± 0.0369
S = 6	0.9057 ± 0.0394	0.9077 ± 0.0377	0.9121 ± 0.0383
S = 7	0.9450 ± 0.0285	0.9401 ± 0.0319	0.9388 ± 0.0285
S = 8	0.8887 ± 0.0366	0.8962 ± 0.0335	0.8939 ± 0.0362
S = 9	08825 ± 0.0452	0.9226 ± 0.0364	0.9202 ± 0.0384
Average	0.8975 ± 0.0399	0.9037 ± 0.0377	0.9037 ± 0.0382

*AUC* Area under the curve of receiver operating characteristic curve, *LMA* Learning model by multiple angles

The best performing model follows the same convention from the previous model with an AUC of 0.9450 for angle = 13 and S = 7. However, the average AUC tells us that this angle group is not stable with the stride varying, as shown in Tables 6 and 7. Considering the stability of the model, the group with 62 angles shows some advantages over others. Its averaged AUC score reaches 90.55% with the smallest standard deviation 0.0378. The results of 62 angles also indicate that the 1st and 2nd nearest neighbors contain more distinctive texture descriptors while the third nearest neighbor brings redundant information which hurts the classification performance to a small extent. Compared to the first row in Table 5, the AUC scores improved about 1%–2% with increasing directions. The multi-stride continues enhancing this criterion to 94% when s = 7.

### Comparisons

To illustrate the efficiency of our method, some typical methods are introduced to compare with our models. These methods are listed as the following.

- HF – a typical method was proposed to construct the texture descriptor consisting of 28 HFs extracting from CM [15].
- eHM – a new model introduced 30 measures to represent texture characteristics extracted from CM [23].
- K-L transform based eHM (eHM + KLT) – this method introduced K-L transform to enhance the distinction between two different image features and reduce variation [23].
- Co-occurrence of local anisotropic gradient orientation (CoLIAGe) – this model employed gradient angles and extracted the entropy of every local patch to form a global texture descriptor by two joint histograms [33].
- VGG16 – this method extracts 20 salient slices from every polyp volume to feed to VGG16 for polyp classification [34].

**Fig. 8:**
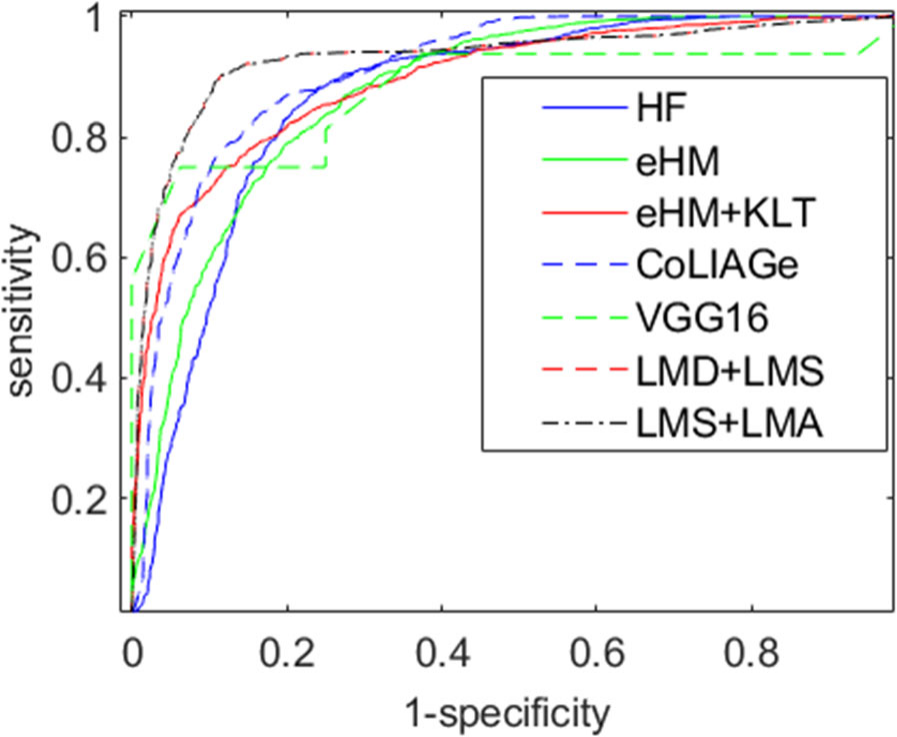
Receiver operating characteristic curves of six methods via random forest except VGG16. HF: Haralick feature; eHM: Extended Haralick measure; KLT: Karhunen-Loeve transform; LMD: Learning model by multiple displacements; LMS: Learning model by multiple strides; LMA: Learning model by multiple angles; CoLIAGe: Co-occurrence of local anisotropic gradient orientations

We choose two results from two hybrid results of our method for this comparison, i.e., LMD + LMS with stride = 7 and D = 1, LMS + LMA with angles = 62 and stride = 7. Their receiver operating characteristic curves are plotted in Fig. 8 which illustrates their different performances for polyp classification. Moreover, their AUC scores, accuracies, sensitivities and specificities are also listed in Table 8 for further evaluation. To verify their differences, *P*-values are calculated using t-test to determine if our method is significantly different from others, as shown in Table 9. All the P-values are far smaller than 0.05 which indicates that the proposed methods are distinctive to all the typical methods.

**Table 8:** Four evaluation measurements for seven methods

Method	AUC	Accuracy	Specificity	Sensitivity
HF	0.8751	0.8151	0.8093	0.7693
eHM	0.8863	0.8363	0.8281	0.7256
eHM + KLT	0.9073	0.8873	0.8812	0.8475
CoLIAGe	0.9229	0.8835	0.8393	0.8331
VGG16	0.8234	0.8404	0.8069	0.8066
LMD + LMS	0.9449	0.8934	0.9019	0.8851
LMS + LMA	0.9447	0.8915	0.8801	0.9031

*HF* Haralick feature, *eHM* Extended Haralick measure, *KLT* Karhunen-Loeve transform, *LMD* Learning model by multiple displacements, *LMS* Learning model by multiple strides, *LMA* Learning model by multiple angles, *CoLIAGe* Co-occurrence of local anisotropic gradient orientations, *AUC* Area under the curve of receiver operating characteristic curve

**Table 9:** Wilcoxon signed-rank test between AUC scores of our methods and the typical methods

Our method	HF	eHM	eHM + KL	CoLIAGe	VGG16
LMD + LMS	<< 0.05	<< 0.05	<< 0.05	<< 0.05	<< 0.05
LMS + LMA	<< 0.05	<< 0.05	<< 0.05	<< 0.05	<< 0.05

*HF* Haralick feature, *eHM* Extended Haralick measure, *KLT* Karhunen-Loeve transform, *LMD* Learning model by multiple displacements, *LMS* Learning model by multiple strides, *LMA* Learning model by multiple angles, *CoLIAGe* Co-occurrence of local anisotropic gradient orientations, *AUC* Area under the curve of receiver operating characteristic curve

## Conclusions

This paper reviews the properties and evaluates the potential of the Haralick model by examining several weaknesses observed in practice [22, 23]. The multi-scale gray level co-occurrence matrix (MSGLCM) is proposed and aims to improve this model by incorporating the multi-scale analysis technique with GLCM to evaluate the three variables: displacement, stride, and angular directions. MSGLCM combines the stride and down-sampling technology to emphasize the unique features and lessen the number of non-critical characteristics to improve polyp classification performance. Meanwhile, MSGLCM adopts up-sampling techniques to integrate the displacement and angle to get new texture patterns which could mitigate the sparse sampling problem within the GLCM calculation. With the increase of texture patterns and texture descriptors, the forward step feature selection method is applied to solve the informative redundancy and overfitting issues in polyp classification over 63 polyp masses: including 32 invasive adenocarcinomas and 31 benign adenomas.

Experimental results reveal that increasing stride can significantly improve polyp classification over the traditional HF and eHM. On the other hand, displacement has little if any positive effect on the results on its own. With the addition of increasing displacements whilst preserving the lower displacements, there were varying results which demonstrates that there can be potential gains in additional displacements. This proposed model can achieve higher AUC values compared to the typical methods discussed in section 3.3. The best model from our experiments had a 6.23% improvement and reduced the standard deviation by 34.95% which is a significant advantage over them.

## Discussion

Why the stride is more sensitive than the displacement and angle is still a question for us. The reasons might be guessed from two aspects. The type of polyp texture might be the first reason. The polyp texture should belong to one type of stochastic texture which has no apparent textural structures [35–37]. This type of texture is not sensitive to the changes of directions and displacement because of its isotropy. The second reason might be informative redundancy introduced by multiple angles on stochastic texture. The multi-angle sampling produced too many similar texture patterns from polyps. Since the classification and recognition should depend on some unique features which should play a key role in it, these unique features always make up a very small proportion in the whole feature space [38]. The traditional full-sampling or up-sampling technique makes its proportion much smaller. The stride seems to lessen the non-critical texture patterns and improve the ratio of unique features while down-sampling. We found that the higher sampling rate from a low stride may drown out those texture patterns that are necessary for distinguishing between pathologies.

In summary, our proposed model has shown encouraging performance. Nevertheless, redundant information and over-fitting issues by descriptor variables still face great challenges which need new feature selection technologies to solve. Further investigation is required in implementing convolutional neural networks to solve polyp classification on a small database [25]. Both of these are important tasks for our research efforts in the future.

## Data Availability

All experiments are performed on a private database of IRIS (Imaging Research and Informatics) Laboratory, State University of New York at Stony Brook.

## Abbreviations

2D: Two-dimensional
AUC: Area under the curve of receiver operating characteristic curve
CADx: Computer-aided diagnosis
CM: Co-occurrence matrix
CoLIAGe: Co-occurrence of local anisotropic gradient orientations
CRC: Colorectal carcinoma
CTC: Computed tomographic colonography
DT: Decision tree
eHM: Extended Haralick measure
GLCM: Gray-level co-occurrence matrix
HF: Haralick feature
KLT: Karhunen-Loeve transform
LMA: Learning model by multiple angles
LMD: Learning model by multiple displacements
LMS: Learning model by multiple strides
MSGLCM: Multi-scaling gray-level co-occurrence model
OC: Optical colonoscopy
RF: Random forest
ROIs: Regions of interest
VOI: Volume of Interest
